# A Dictionary of Practical Medicine. Part X

**Published:** 1846-01

**Authors:** 


### A Dictionary of Practical Medicine. Part X. By *James Copland,* M.D. F.R.S. 8vo. pp. 144. 1845.

As we intend reserving any remarks we may have to make upon the mode in which the author of this gigantic undertaking has fulfilled his task, until we have the whole of the work before us, we should have passed the present Part by with the simple remark, that it equals its predecessors in the amount of valuable matter condensed into a small compass, and exceeds them in the number of accompanying original observations; but that we find some of the remarks upon the pathology of Paralysis, and the nature of Cholera, of too interesting a character to be thus summarily dismissed. To these then we will briefly refer.

### Of certain Points in the Pathology of Paralysis.

Palsy may be induced by whatever interferes with the generation or transmission of the nervous power. Thus, when the grey substance of the brain and spinal chord, which is the portion chiefly instrumental in the production of the peculiar functions of these organs, becomes diseased, palsy is found to prevail in proportion to the extent in which it does so, as is seen especially in cases of general paralysis complicating insanity. But the nervous power may be duly generated, and yet its transmission through the frame in part impeded, whether by injury, disease, or compression; while, in other cases, due evolution and transmission are alike prevented by disease which implicates both grey and white substances.

*Crossed Paralysis.*—Dr. Copland agrees with Dr. Bennett in doubting the accuracy of the observations which have been adduced to show the occasional occurrence of paralysis on the same side as the lesions in the brain. Twenty-one such cases, one-half of which will not bear scrutiny, only are recorded.

"Numerous instances have occurred of abscesses, softening, and other alterations of the brain having been found, but in which no paralysis had been observed during life; and a still greater number are on record, in which there was well-marked paralysis, but no appreciable lesion of structure after death. It is by no means improbable, therefore, as paralysis may be induced without leaving any traces, that, in those few cases where the palsy and lesion in the brain were on the same side, it was really caused by undetected changes in the opposite hemisphere; and, as is sometimes the case, that the disease found in the hemisphere of the paralysed side had not occasioned the loss of motion."

Lesions in the spinal-marrow, however, produce a direct effect. Dr. Copland is disposed to view the extensive changes in this organ which are sometimes found at the autopsy (such amounting even to a complete destruction of the chord, the patient still retaining the power of voluntary motion to the last), as having frequently commenced shortly before death and been completed subsequently to that event. The involuntary or automatic movements produced by an inflamed spinal chord may be mistaken for voluntary ones, and reflected motions may occur where all means of transmitting ordinary volition is destroyed.

The condition of the *cerebro-spinal fluid* must not be lost sight of in estimating the effects of lesions in producing paralysis. Its protective agency in the spinal canal, where it is abundant, has been shewn by Magendie and Todd; and its quantity doubtless varies with the vascular condition of the nervous matter and membranes.

"It may reasonably be concluded that, when these structures, and the blood supplying them, do not sufficiently fill the unyielding cases of the spine and the brain, the fluid interposed between the arachnoid and pia-mater will supply the defect, and prevent the existence of any vacuum; and that, on the other hand, when the states of these centres and of the circulation in them are such as give rise to much fulness, the quantity of this fluid will be diminished. Anæmia will thus be attended by an increase of the cerebro-spinal fluid, and vascular turgescence by a diminution of it; the included masses being thereby preserved from much diminution of pressure in the one case, and from much increase of it in the other. Thus, also, in cases of atrophy, partial or general, of the brain or spinal chord, the quantity of this fluid is increased, showing the importance of it to the functions of these parts, whilst in cases of hypertrophy it is diminished or almost wanting.

"From what I have now adduced it may be inferred, that the effects often imputed to the abundance of this fluid, particularly in the spinal canal, by several pathologists, when detailing the morbid appearances after death from diseases of the nervous system, have been imputed to a wrong source; that the serous effusion in these cases, as I have elsewhere argued, is neither the cause of pressure upon, or of induration of, the nervous centres, nor the source of the palsy sometimes observed, but that it is a result of those changes of the nervous structure, and of the local circulation with which it is found associated, in connection with, or aided by, the unyielding state of the surrounding parts."

*Influence of the different columns of the spinal medulla and of the roots of the spinal nerves upon the sensitive and motor powers.*—The exclusive devotion of the posterior columns of the chord to the conveyance of sensation is by no means so satisfactorily made out, as is that of the anterior columns for the transmission of volition. Extensive lesions of these posterior columns recorded by several authors, have either occurred subsequently to death, or prove that sensations may be conveyed by other channels, independently of these columns. The sympathetic system seems to be this other channel.

"When we recollect that communicating branches run between the ganglionated or posterior roots of the nerves and the great sympathetic on each side; that ganglial nerves may be traced in their course from the sympathetic into the spinal ganglia and chord on the one hand, and from the latter into the sympathetic and ganglia on the other, we cannot but infer, not only that sensation may be transmitted, or more correctly, that impressions on the surface may be conveyed to the brain so as to excite consciousness, by a different route than that of the spinal chord, especially under circumstances of gradual change in the chord, rendering it ultimately incapable of discharging this function, and that this other route is through the sympathetic nerves, and their communications with the posterior roots of the nerves and spinal medulla. * * * The above considerations may serve as reasons wherefore sensation remains unimpaired, or but little affected, in very many cases where the chord is diseased or injured so as to be incapable of transmitting the impulse of volition, particularly when the lesion is high in the chord, and when it has advanced slowly or gradually. They may also account for the rare occurrence of entire loss of sensation in any form of palsy of motion."

*Congestion of the venous sinuses seated between the theca of the chord and the bodies of the vertebræ.*—Dr. Copland observes, that the pathological relations of congestion and obstruction by coagula of these sinuses have been neglected. Causes producing defective innervation or vascular determination to the chord, induce congestion of the sinuses, which, in its simplest state, produces pain or weakness of the back and lower extremities, and sometimes incomplete palsy of motion in the latter; and, when it is variously associated with pain, may be mistaken for rheumatism, neuralgia, or gout. Continued congestion eventually produces a distension of the capillaries and serous effusion, which may induce pressure on the roots of the nerves and consequent paralysis.

"Congestion of the spinal sinuses, with more or less of the consequences now mentioned, is a frequent attendant on *fevers*, particularly the more adynamic and congestive forms, occasioning not merely pains and weakness of the back and limbs and incomplete palsy of motion of the lower extremities, but also more or less affection of the urinary organs. Many of the cases described as spinal irritation, of hysterical neuralgia, of uterine irritation, &c. actually are instances of congestion of the spinal sinuses, occasioning remote or sympathetic phenomena in addition to those which are more strictly local. These are often removed, or partially relieved, for a time by the natural recurrence of the catamenia; but, when more extensive or severe, or when associated with suppression of this discharge, they sometimes lapse into paraplegia or partial palsy, especially when neglected or injudiciously treated, owing to an increase of the congestion or of its consequences."

*Mechanism and Functions of the Spinal Chord.*—Dr. Copland states that, in the articles Cholera, Chorea, and Convulsions, he explained the symptoms of these diseases as arising from reflex actions excited in the voluntary muscles by irritations transmitted to the roots of the spinal nerves and spinal chord. Dr. M. Hall has subsequently referred these and analogous phenomena to a special organization of the spinal chord. Of this explanation, as applied to the convulsive movements excitable in the palsied, Dr. C. thus observes :—

"Such being the mechanism of ordinary sensation and motion, according to the recent researches of Stilling, Van Deen, Budge, and others, it can be no longer difficult to account for those involuntary movements which are produced in a paralysed limb when the surface of it is irritated, pinched, or tickled, and which have been termed by Dr. M. Hall reflex actions, depending, according to him, upon a reflex function of the spinal chord, which function he refers to a distinct mechanism of the chord. It has already been contended by the author that no such mechanism exists, and that these actions are sympathetic, and result from the conformation of this part of the nervous system, transverse fibrils, passing, as shown by the anatomists just referred to, directly from the posterior to the anterior grey substance, to convey impressions from the sensitive fibrils and to excite the roots of the motor nerves. That no appropriate and peculiar structure exists in the chord for the purpose of performing these sympathetic or reflex movements, beyond what has now been noticed, is the opinion not only of the author, but also of the writers already mentioned, as well as of many others who have investigated the subject."

*Relaxation of the Sphincters.*—This is not of so general occurrence as usually believed. They "are not so frequently relaxed as they are imperfectly influenced by the will, or are not at all affected by it." The tonicity of these muscles has been erroneously supposed to be dependent upon the spinal chord to the neglect of the ganglial system, which is its real regulator.

"Pathological evidence, indeed, clearly leads to these inferences, 1st, that the power of the sphincters is attributable chiefly to the organic nervous system, but that it is increased by volition exerted through the medium of the spinal nerves, especially in circumstances requiring such increase, as when the disposition to the action of the bowels or the bladder has to be resisted; and 2nd, that it is chiefly this latter influence, or that which is exerted through the spinal chord, that is either lost or impaired, in cases where the voluntary contractions of the sphincters are insufficient to prevent the passage of the excretions when the patient wishes to retain them. It is not, therefore, to be inferred that, where there is insufficient control over the evacuations, the sphincters are either relaxed or materially deficient in power; but that they are only insufficiently influenced by volition, relatively to the power which overcomes their natural tonicity."

### Pestilence (Choleric).

In the article thus headed we are furnished with an able and minute description of the disease commonly, but erroneously, termed *Epidemic* or *Indian Cholera*. The conclusions which Dr. Copland has arrived at are entitled to the gravest consideration, formed, as they have been, from ample opportunities of observing the disease during its visitation, and an elaborate examination of numerous reports and documents placed at his disposal by the East India Company. The devastating nature, and wide-spread influence of the scourge entitle it to be placed, together with the Plague and Yellow Fever, in the category of Pestilential Diseases; and Dr. Copland first refers to the several erroneous views which extensively prevail respecting the nature of the malady. 1st. It has been very generally supposed that this disease is merely an epidemic form of the common spasmodic cholera of warm climates. Dr. C. had had opportunities of treating the spasmodic cholera in insalubrious climates, and, so long ago as 1822, protested against the two diseases being confounded together. In India, too, where spasmodic cholera was well known, when this disease first appeared, it was looked upon as a new, unheard-of, pestilence. Certain by no means essential symptoms, the purging, vomiting, and spasms, have been mistakingly set down as characteristic ones. 2ndly. An opinion has been erroneously held and mischievously propagated in this and other countries, that the disease did not exhibit proofs of contagion in the East. 3rdly. It has been, and is very generally, believed that the disease is produced by some unknown condition of the air. It would be difficult to explain its uninterrupted duration for near thirty years by any such supposition, although there can be no doubt it may become greatly aggravated by the prevalence of certain conditions of the atmosphere.

"We know that some diseases are simply infectious without being epidemic, that others are both infectious and epidemic, and others are epidemic and only contingently infectious. But the author believes that, like eruptive and typhoid fevers, this distemper is infectious, is not essentially epidemic, although it will, during favourable states of the atmosphere, &c. assume epidemic characters, and be modified accordingly. An attentive review of the various modifications of the malady in India, throughout Asia, in Europe, and in America, seems to justify this view, and to confirm the conclusion as to its being a specific disease, arising from a specific cause, but promoted and disseminated more widely by the aid of various concurrent causes, amongst which, epidemic or unhealthy constitutions of the air; dirty, crowded, and close apartments; and crowding of the sick, are the most prominent."

After detailing the various diagnostic marks by which this disease is distinguished from *cholera* properly so called, Dr. Copland observes:—

"After attentively considering its phenomena and nature, I would conclude, 1st. That this malady, as respects the causes which occasion it, and as regards the pathological states which constitute its various grades or stages of intensity, is quite distinct from all the forms of cholera, whether the common *bilious* variety, or the more severe form, usually denominated *spasmodic*, the *mort-de-chien*, &c.; and that, therefore, the name cholera should be discarded from all scientific descriptions of it. 2ndly. That the accounts which we possess of the epidemics and pestilences which have ravaged various countries in former times, do not furnish us with the history of any disease which may be considered as identical in its nature with this pestilence; and that it must, owing to this circumstance, and to the uniformity of its characteristic phenomena, be viewed as being of modern origin and *sui generis*. As it is important that the name of a disease should not be such as may risk its being confounded with another, different from it in nature, symptoms, and termination, so I consider that some other name than that at present applied to it should be given it. As to the particular appellation that may be employed, I conceive that one pointing to its chief pathological states and its prominent tendencies, ought to be preferred. The intense influence of its exciting causes upon all the respiratory actions and functions, as well as upon the actions of the heart and state of the pulse, and its marked tendency to propagate itself, and to terminate fatally, have induced me to apply to it the name of *Asphyxia pestilentia,* or *pestilential asphyxia.*"

*Infection.*—Dr. Copland observes, that he commenced his investigation of the documents transmitted from India relative to the cholera, in a frame of mind quite unbiassed, and arrived at the conclusions, "that the disbelief of infection was not general in India,—that the productions which issued from the Medical Board very strongly favoured, and indeed proved, the existence of this property,—that two out of the three actually insisted upon the activity of its influence,—and that, therefore, the dangerous opinion, so very generally propagated, and even acted upon, both in this and foreign countries, that the Authorities in India did not consider the disease infectious, is entirely without foundation in truth."

Numerous extracts are furnished, in which facts are stated, even by non-contagionists, which prove the infectious nature of the disease. The question is gone into at length also in reference to the subsequent progress of the disease in Europe, when its nature and manifestations continued precisely the same, however much it may have varied in intensity. The conclusion irresistably arrived at is, that the disease has never shewn itself "without the presence of a certain leaven, or morbific matter, which, emanating from the bodies of the affected, and floating in the air, is respired by those about to be attacked." Like other diseases, avowedly contagious, it spared many exposed to its influence, its attack being determined by various predisposing, concomitant, and determining causes. The arguments of the non-contagionists are examined at length, on account of the immense practical consequences, as regards prevention of extension, which must necessarily arise from the decision arrived at, and because the same arguments are applicable to the question of the contagion of the plague and yellow fever. From his observation of the disease as it occurred in our own country, Dr. Copland draws the following conclusions:—1. The distemper was manifestly infectious. 2. It was not propagated by contact, but by an effluvium or miasm emanating from the person of the diseased, and inspired by the bye-stander, who became liable to be affected in proportion to his predisposition. 3. This emanation was often manifest to the senses of smell and even of taste, and became attached to articles of clothing, &c., so as to be capable of producing the disease long afterwards. 4. In this way, the disease was frequently propagated by the clothes of the physician and others, who did not themselves become affected. 5. Placing the hand in contact of the surface of a person suffering under the cold stage often produced a peculiar tingling sensation; but did not cause infection, if breathing the contaminated air were avoided. 6. When such air was breathed for the first time by a healthy person, a morbid sensation was often referred to the chest and epigastrium, giving rise to frequent and forcible inspiration. Such impression and its consequences usually disappeared if recourse was had to stimuli and full living, but were followed by other grades of the disease under the influence of various depressing agents. 7. Subsequent exposure to infected air, unless this effluvium were more concentrated, usually produced less manifest morbid impressions. 8. The operation of the effluvium was violent in proportion to its concentration, and the debility and degree of predisposition of the person respiring it. 9. "There is no evidence to account for the generation of the choleric poison in the first instance, and there is as little of its reproduction *de novo*, on subsequent occasions. It is also impossible to form a correct idea of the period during which the infectious miasm or seminium may be retained by clothes closely shut up from the air, or by the dead and buried body, and be still capable of affecting the healthy."

We forbear expressing, at the present time, any opinion of the justness of Dr. Copland's conclusions, intending, at no distant period, to bring the whole subject of infection and quarantine under the notice of our readers. In the mean time, however, we have thought it right to re-produce the opinions of so deservedly high an authority upon a question which is so anxiously occupying the attention of the various European governments. Dr. Copland is strongly of opinion that, to the neglect of precautionary measures on the appearance of this fatal disease in India, is due its extensive prevalence over so large a portion of the globe.

Although the extract is rather a long one, we must find space for the following able summary.

"I conclude this part of the inquiry by stating the inferences which may be drawn from an extensive view of what is known of this pestilence, as it has appeared in Asia and in Europe, and from intimate observation of its phenomena, as they lead to various considerations calculated to arrest its progress and to remedy it, when an attack has not proceeded too far in the destructive processes in which it has been shown to terminate. A. The pestilential cholera seems to have been propagated by an animal miasm or effluvium of a peculiar kind, emanating from the bodies of the affected; and this effluvium being inhaled with the air into the lungs, paralyses these organs, and acts as a poison on the class of nerves which supplies the respiratory, the assimilating, the circulating and secreting viscera, vitiating also the whole mass of blood, and thereby occasioning a specific disease, which in its turn gives rise to an effluvium, similar to that in which itself originated; which, also, in like manner, perpetuates its kind, under the favorable circumstances of predisposition, aerial vicissitudes, &c., and thus a specific form of disease is propagated far and wide, as long as predisposing, concurrent, and determining causes favour its propagation. B. The morbid impression of this effluvium or poison upon the nerves of organic life, and probably the effects of its introduction into the current of the circulation, are of a sedative kind, rapidly destroying the vital energy of the former, and vitiating the latter, and thereby giving rise to the characteristic phenomena of the malady. C. The impression of the effluvium on the organic class of nerves, and the vitiated state of the blood, may be viewed as the proximate cause, not only of the disturbance evinced by the respiratory, the secreting, the assimilating, and the circulating functions, but also of the morbid actions of the stomach and bowels, and the copious serous discharges from these organs, as well as of the muscular spasms, the sinking of all the vital and animal powers, of the shrunk and collapsed state of the surface of the body, of the black, thick state of the blood, and of the rapid depression of the animal temperature. D. The states of the perspiration and skin, and the discharge of the serous portion of the blood by the stomach and bowels, imparting the peculiar appearance of the evacuations, proceed from the alteration primarily produced in the vitality of the frame, and in the condition of the blood; and it is chiefly through the medium of the cutaneous surface, of the liver, of the kidneys, and of the mucous membranes, assisted, perhaps, also by the other secreting viscera, that the morbid change of the blood is remedied, and impurities removed from it. E. The advanced stages, or the consecutive, or febrile symptoms of the disease, whether those chiefly depending upon the state of the nervous functions, or of the circulation within the brain, or proceeding from the condition of the abdominal viscera, arise partly from the shock received by, and the depression of, the vital energy of the frame in the early stage, partly from the congested condition of the large veins and important viscera, and partly, if not chiefly, from the alterations which had taken place in the blood during the early stages of the malady. F. The effluvium or seminium, which propagates the distemper, is generated in the progress of the changes produced in the blood, and is emanated or discharged from the mucous surfaces of the lungs and digestive canal, and from the cutaneous surface, along with their respective exhalations and excretions; and this seminium, by contaminating the surrounding air, or woollen cloths and animal products, capable of attracting and retaining for awhile animal effluvium, affects those of the healthy who are predisposed, either constitutionally, or by antecedent, concomitant, or determining influences, or on whom this efficient agent acts in an intense or concentrated form, or is aided by accessory or concurrent causes."

